# Differences of the 6N and 6J Substrains of C57BL/6 Mice in the Development of Experimental Autoimmune Encephalomyelitis

**DOI:** 10.1002/mco2.70228

**Published:** 2025-07-02

**Authors:** Ana Isabel Álvarez‐López, Eduardo Ponce‐España, Ivan Cruz‐Chamorro, Guillermo Santos‐Sánchez, Ignacio Bejarano, Nuria Álvarez‐Sánchez, Patricia Judith Lardone, Antonio Carrillo‐Vico

**Affiliations:** ^1^ Instituto de Biomedicina de Sevilla IBiS/Hospital Universitario Virgen del Rocío/CSIC/Universidad de Sevilla Seville Spain; ^2^ Departamento de Bioquímica Médica y Biología Molecular e Inmunología Facultad de Medicina Universidad de Sevilla Seville Spain

**Keywords:** C57BL/6J, C57BL/6N, multiple sclerosis, microbiota, mouse model, neuroinflammation

## Abstract

Multiple sclerosis (MS) is an autoimmune disease causing neuroinflammation and demyelination in the central nervous system (CNS). It is traditionally considered CD4^+^ T cell‐mediated, but several immune cells, such as CD8^+^ cells, B cells, macrophages, and dendritic cells (DC) also contribute to the pathogenesis. Moreover, altered gut microbiota, including changes in specific genera, has been observed in MS patients. The murine model of MS, experimental autoimmune encephalomyelitis (EAE), is mainly carried out in C57BL/6 mice. Historically, N and J substrains have been used interchangeably, and many laboratories are not even aware of which strain they are using. Therefore, the objective of this study was to evaluate the differences between the 6J and 6N substrains subjected to myelin oligodendrocyte glycoprotein (MOG_35–55_) induced EAE in the composition of neuroinflammatory cells and microbiota. 6J substrain presented a more severe EAE than the 6N substrain, accompanied by an increase in the frequency of macrophages, CD8^+^, and B cells within the infiltrated immune cells compartment. In addition, 6J animals have a higher proinflammatory profile and a lower anti‐inflammatory profile compared with the 6N substrain. Consistent with this, the differences observed in the basal microbial taxa between both substrains support the differences observed in the immunological response.

## Graphical abstract

The 6J substrain exhibits more severe EAE than 6N, with increased macrophages, CD8^+^, and B cells in the CNS, as well as a higher proinflammatory profile and lower anti‐inflammatory profile, correlating with differences in microbial taxa.

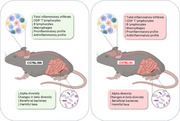

## 1 Introduction

Multiple sclerosis (MS) is an autoimmune disease characterized by inflammation and demyelination in the central nervous system (CNS) with a prevalence of 35.9 cases per 100,000 individuals [1]. Symptoms vary from person to person and may include mobility challenges, disabling fatigue, vision problems, and cognitive changes. Currently, there is no cure; therefore, early diagnosis and treatment are critical to minimize disability [2]. The cause of the disease is unknown, but it is known to involve a combination of genetic susceptibility and other factors, such as the environment or having an Epstein–Barr virus infection [3, 4]. Classically, MS is regarded as a T‐cell‐mediated autoimmune disorder with a predominance of CD4^+^ cells [5, 6, 7]. However, the involvement of various immune cell types that also infiltrate the CNS, such as CD8^+^ and B lymphocytes, macrophages, and dendritic cells (DC), has also been shown to be involved in the pathogenesis of the disease [8]. Specifically, inflammation‐induced axonal lesions begin with excessive production of TNF, IFN‐γ, and IL‐17 proinflammatory cytokines by the CD4^+^ and CD8^+^ T lymphocytes [9, 10]. B cells and macrophages also contribute to TNF production [11, 12]. In addition, pathogenic macrophages in inflammatory MS lesions express specific markers of inflammation, such as inducible nitric oxide synthase (iNOS) [13]. In contrast, there are regulatory populations that aim to counteract this process in the CNS, such as regulatory T cells (Treg), regulatory B cells (Breg), and the classically named M2 macrophages [14, 15], characterized by the expression of arginase 1 (Arg1). In addition, IL‐10 has a very important anti‐inflammatory role. In fact, high levels of this cytokine correlate with an improvement in the state of the disease [16].

Previous studies have reported that the etiopathogenesis of MS is also strongly affected by diet and therefore by gut microbiota [17, 18]. Indeed, MS patients have been shown to have altered gut microbiota compared with healthy people [17, 19, 20]. Specifically, they have a decrease in the genera *Prevotella* and *Parabacteroides* and an increase in *Alistipes* and *Bacteroides*, among other changes [19, 21, 22]. Moreover, certain species are associated with a relapse or worsening of the disease, suggesting that microbial factors could potentially exacerbate neuroinflammation and symptoms of MS [23]. Consequently, the number of studies analyzing the brain‐gut axis in the context of this disease has recently increased [24].

MS is classified into four different clinical forms, with relapsing–remitting MS (RR‐MS) initially diagnosed in more than 80% of all patients [25]. The animal model most used for the study of MS is the experimental autoimmune encephalomyelitis (EAE), which simulates a typical inflammatory exacerbation of the RR‐MS, and it is carried out in C57BL/6 mice. In this model, animals develop a disability that begins in the caudal area and increases toward the rostral area. A clinical score is assigned to evaluate the disease severity so that a higher score means a greater severity of the animal [12]. Different substrains derived from the C57BL/6 strain have been described based on the genetic and phenotypic changes. Among them, the C57BL/6J (6J) and C57BL/6N (6N) substrains are the most widely used in the biomedical scientific community [26]. Although there are previous studies that show differences between the 6J and 6N substrains in terms of cardiovascular, metabolic, and neurological diseases [27], there is no evidence related to the development of EAE. In addition, studies related to differences in the basal gut microbiota between 6N and 6J substrains and how these could affect the development of EAE are completely unknown. Therefore, the objective of this study was to evaluate the differences in the neuroinflammation associated with EAE between the 6J and 6N and the role of the microbiota as a predisposing factor to the differences in EAE.

## 2 Results

### 2.1 The 6J Substrain Develops More Severe EAE Than the 6N Substrain

**FIGURE 1:**
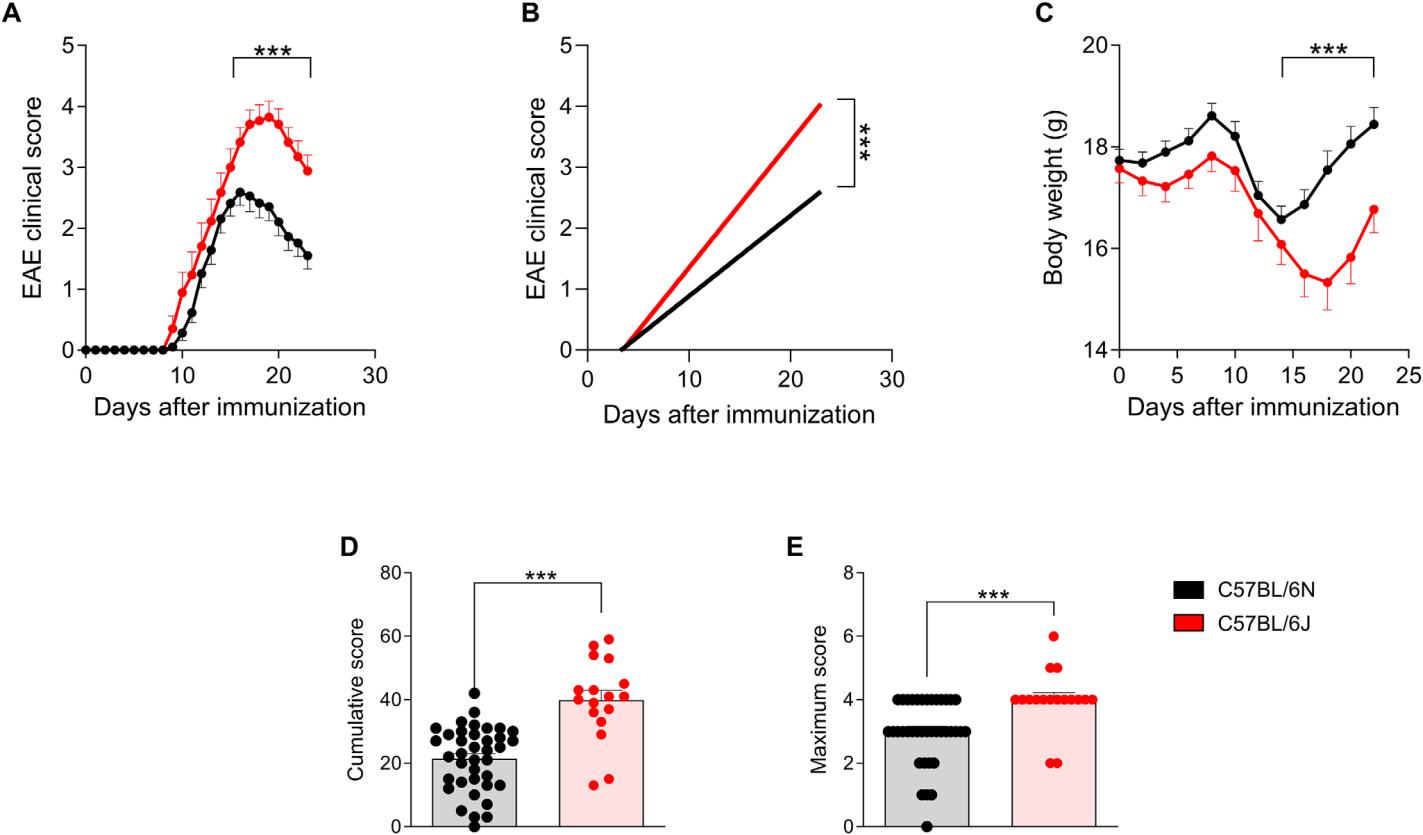
EAE disease course in C57BL/6N and C57BL/6J strains. EAE clinical scores (A), regression curves (B), and body weight (C) of the animals during the experiment. Cumulative (D) and maximum (E) clinical scores that were reached by each animal in both groups on day 23 postinduction. Each dot represents an individual animal. Bars represent the mean ± SEM of C57BL/6N (*n* = 39) and C57BL/6J (*n* = 17) mice. ***p ≤ 0.001.

The present results revealed significant differences in the severity of EAE between the two substrains, highlighting the greater susceptibility of the 6J substrain compared with the 6N. During the course of the disease, 6J mice showed markedly more pronounced clinical signs, as evidenced by the higher clinical scores recorded over time (Figure 1A). This increase in severity was not only reflected in daily clinical evaluations but was also corroborated by cumulative disease scores, where 6J mice showed significantly higher values compared with their 6N counterparts (Figure 1B,D). Similarly, the maximum clinical signs observed at the peak of the disease were considerably more severe in the 6J substrain (Figure 1E).

Additionally, body weight measurements revealed that 6J mice experienced a more pronounced weight loss, a hallmark of disease progression in EAE, compared with the 6N substrain (Figure 1C). This observation further supports the conclusion that the 6J substrain is inherently more susceptible to EAE induction under the same experimental conditions.

These findings demonstrate significant substrain‐specific differences in the clinical manifestation of EAE.

### 2.2 Infiltrated Immune Cell Subsets Differ According to the Substrain

**FIGURE 2:**
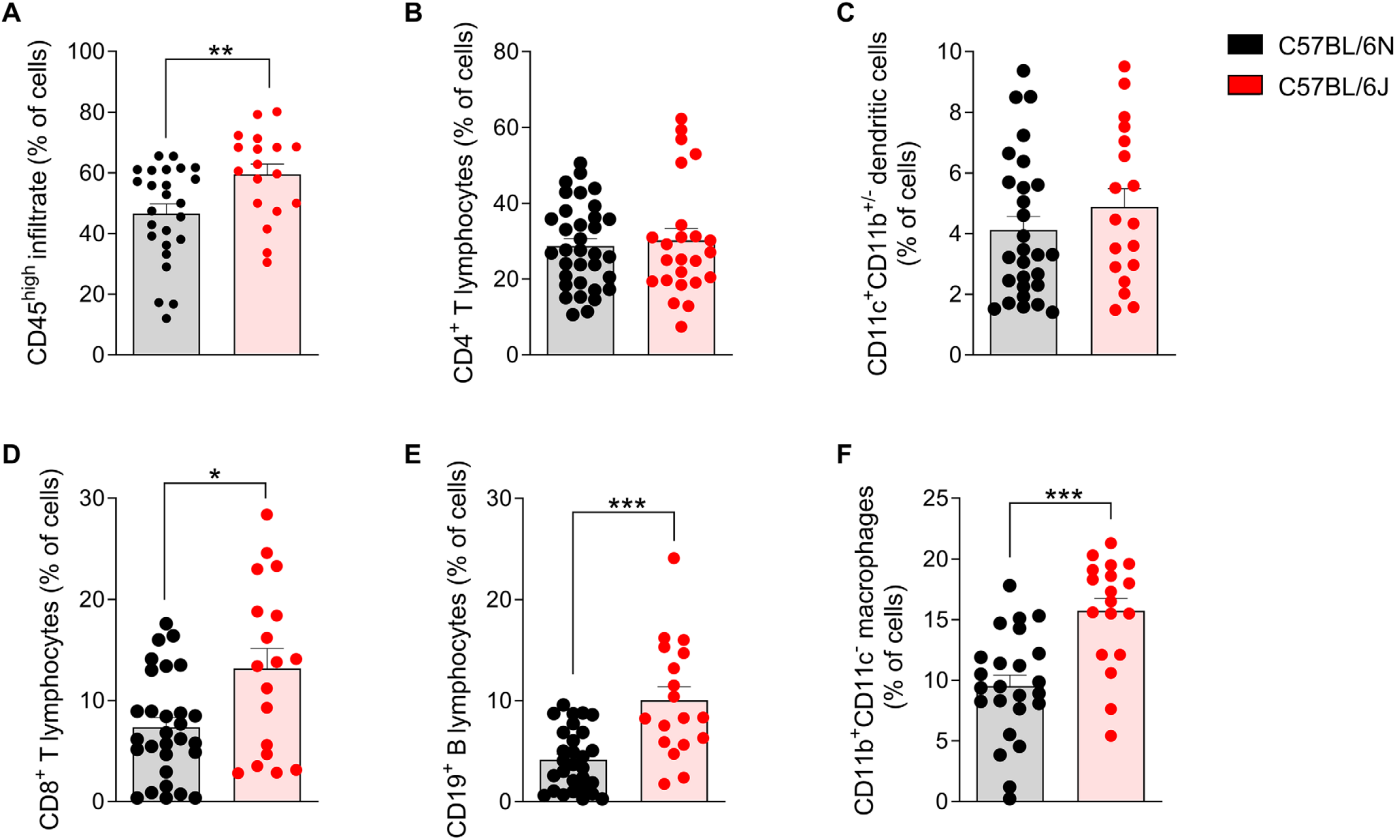
Infiltrating immune populations in the CNS of EAE‐induced 6N and 6J mice. Frequencies of total inflammatory infiltrate (A; C57BL/6N, *n* = 25; C57BL/6J, *n* = 18), CD4⁺ T lymphocytes (B; C57BL/6N, *n* = 34; C57BL/6J, *n* = 24), CD11c⁺CD11b⁺/⁻ dendritic cells (C; C57BL/6N, *n* = 28; C57BL/6J, *n* = 18), CD8⁺ T lymphocytes (D; C57BL/6N, *n* = 28; C57BL/6J, *n* = 18), CD19⁺ B cells (E; C57BL/6N, *n* = 28; C57BL/6J, *n* = 18), and CD11b⁺CD11c⁻ macrophages (F; C57BL/6N, *n* = 28; C57BL/6J, *n* = 18) were determined by flow cytometry. Each dot represents an individual animal. Bars represent the mean ± SEM. *p ≤ 0.05; **p ≤ 0.01; ***p ≤ 0.001. CD, cluster of differentiation; CNS, central nervous system.

The composition of immune cells that infiltrate the CNS of 6J and 6N mice at the peak of the disease was quantified to determine differences related to the progression of EAE. The results showed that 6J mice exhibited significantly higher total leukocytes infiltrating the CNS compared with 6N mice (Figure 2A), suggesting that the 6J substrain experiences a more pronounced inflammatory response in the CNS during the peak of the disease.

Regarding the composition of infiltrated immune cells, no significant changes were observed in the frequency of CD4^+^ T cells and DC between the substrains (Figure 2B,C). These results indicate that, despite the higher leukocyte infiltration in 6J mice, the number of CD4^+^ T cells and DC populations do not seem to contribute differentially to the inflammatory pattern observed between the two substrains. However, significant differences were observed in other immune cell populations. The frequency of CD8^+^ T cells, B lymphocytes, and macrophages increased significantly in the CNS of 6J mice compared with 6N mice at the peak of the disease (Figure 2D–F).

### 2.3 The Effector T Response Mediated by CD4^+^ and CD8^+^ T Lymphocytes Increases in the 6J Substrain, while the Anti‐Inflammatory Response Mediated by CD4^+^ Cells Is Reduced

To investigate the contribution of CD4^+^ and CD8^+^ T cells to the proinflammatory status of the CNS, the production of key proinflammatory cytokines by these T lymphocyte subsets was quantified, including TNF, IFN‐γ, and IL‐17. Furthermore, to gain a deeper understanding of the differences in the anti‐inflammatory profile between the 6N and 6J substrains, the presence of IL‐10‐producing CD4^+^ T lymphocytes and regulatory T cells (Tregs) was analyzed in the CNS at the peak of the disease.

**FIGURE 3:**
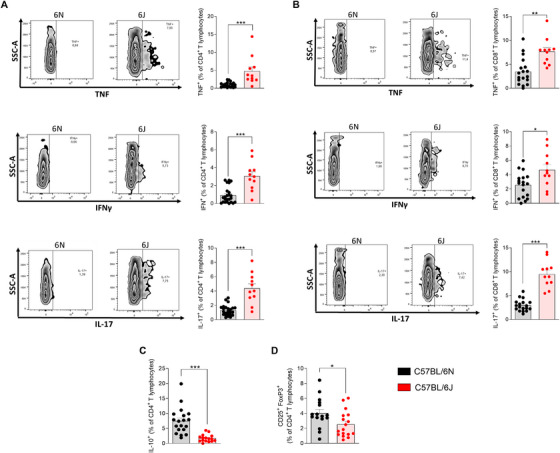
Effector T response mediated by CD4^+^ and CD8^+^ T cells and regulatory T response in the CNS of EAE‐induced 6J and 6N mice. Frequencies of proinflammatory cytokines TNF, IFN‐γ, and IL‐17 produced by CD4⁺ (A; C57BL/6N, *n* = 24; C57BL/6J, *n* = 11) and CD8⁺ (B; C57BL/6N, *n* = 18; C57BL/6J, *n* = 11) T lymphocytes, along with representative flow cytometry plots. Frequencies of CD4⁺ T cells producing IL‐10 (C; C57BL/6N, *n* = 19; C57BL/6J, *n* = 16) and of CD4⁺CD25⁺FoxP3⁺ regulatory T cells (D; C57BL/6N, *n* = 16; C57BL/6J, *n* = 17) are also shown. Each dot represents an individual animal. Bars represent the mean ± SEM. *p ≤ 0.05; **p ≤ 0.01; ***p ≤ 0.001. CD, cluster of differentiation; CNS, central nervous system; FoxP3, forkhead box P3; IFN‐γ, interferon‐γ; IL, interleukin; TNF, tumor necrosis factor.

Specifically, the production of TNF and IFN‐γ by CD4^+^ T lymphocytes was markedly elevated in 6J mice (Figure 3A), indicating an enhanced Th1‐driven immune response in 6J mice, which is consistent with the more severe clinical progression of EAE observed in this substrain. The production of IL‐17 by CD4^+^ T cells was also higher in 6J mice, reflecting an increased Th17 response (Figure 3A).

Similarly, CD8^+^ T cells from 6J mice showed a significantly higher frequency of cytokine production, including TNF, IFN‐γ, and IL‐17 (Figure 3B). Elevated TNF and IFN‐γ point to an increased Tc1 response, while the IL‐17 production supports a more pronounced Tc17 response in these CD8^+^ T cells. These findings suggest that CD4^+^ and CD8^+^ T lymphocytes from 6J mice contribute to the proinflammatory environment of the CNS and play a critical role in exacerbating the autoimmune response.

IL‐10 is a key anti‐inflammatory cytokine known to suppress proinflammatory responses and regulate immune‐mediated tissue damage. The analysis revealed that the frequency of IL‐10‐producing CD4^+^ T lymphocytes was four times higher in the 6N substrain compared with 6J animals (Figure 3C). Additionally, the 6N substrain also exhibited a significantly higher frequency of Treg in the CNS compared with 6J animals (Figure 3D). Treg cells are essential for maintaining immune homeostasis by suppressing excessive inflammation and promoting tolerance.

Taken together, these results demonstrate that 6J mice exhibit an enhanced effector T‐cell response and a diminished suppressive response driving the neuroinflammatory process underlying the pathogenesis of EAE.

### 2.4 The Pro‐/Anti‐inflammatory Balance Associated with B Lymphocytes and Macrophages Is Skewed Toward a Protective Phenotype in 6N Mice

**FIGURE 4:**
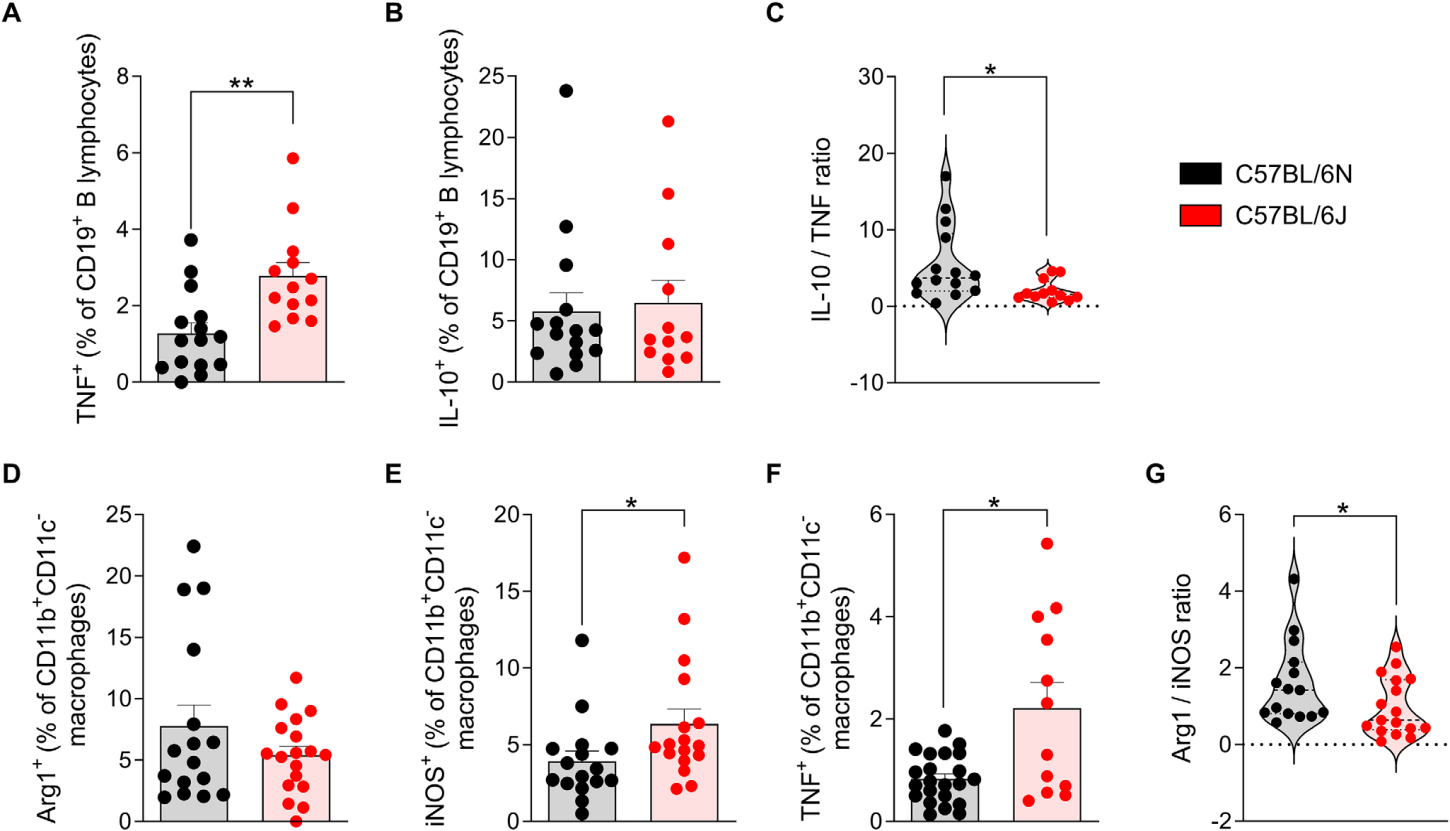
Anti‐/proinflammatory balance in B cells and macrophages in the CNS under the study conditions. Frequencies of TNF‐producing (A) and IL‐10‐producing (B) B lymphocytes in C57BL/6N (*n* = 15) and C57BL/6J (*n* = 13) mice. (C) The ratio between IL‐10⁺ and TNF⁺ B cells. Frequencies of Arg1⁺ (D), iNOS⁺ (E), and TNF⁺ (F) cells among infiltrating CD11b⁺CD11c⁻ macrophages in the CNS at the peak of the disease in C57BL/6N (*n* = 16) and C57BL/6J (*n* = 17) mice for Arg1 and iNOS markers, and in C57BL/6N (*n* = 22) and C57BL/6J (*n* = 12) mice for the TNF marker. (G) The ratio between Arg1⁺ and iNOS⁺ macrophages. Each dot represents an individual animal. Bars represent the mean ± SEM. *p ≤ 0.05; **p ≤ 0.01. Arg1, arginase‐1; CD, cluster of differentiation; CNS, central nervous system; IL, interleukin; iNOS, inducible nitric oxide synthase; TNF, tumor necrosis factor.

To evaluate the role of B lymphocytes in the inflammatory response associated with EAE, the production of TNF and IL‐10 by these cells was analyzed in the CNS at the peak of the disease. TNF production by B cells was significantly higher in 6J mice compared with 6N mice (Figure 4A). Although no statistically significant differences were found in the frequency of IL‐10‐producing B lymphocytes between the two substrains (Figure 4B), the ratio between anti‐ and proinflammatory cytokine production, as reflected by the IL‐10/TNF ratio, was significantly higher in 6N mice compared with 6J (Figure 4C). This indicates that, despite the observed differences in IL‐10^+^ B cells being nonsignificant, the reduction in TNF production in the 6N substrain shifts the balance toward a more protective and anti‐inflammatory phenotype.

Macrophage polarization plays a pivotal role in shaping the inflammatory milieu during EAE, with formerly referred M1 macrophages promoting proinflammatory responses and M2 macrophages contributing to tissue repair and resolution of inflammation. To better understand the differences in macrophage profiles between the 6J and 6N substrains, we assessed the expression of key markers indicative of M1 (iNOS) and M2 (Arg1) phenotypes.

The frequency of macrophages expressing Arg1 in the CNS did not differ significantly between both substrains (Figure 4D). However, 6J mice showed a markedly higher proportion of iNOS^+^ (Figure 4E) and TNF^+^ (Figure 4F) macrophages, indicative of a dominant polarization toward proinflammatory macrophages, compared with 6N mice. Consequently, the Arg1/iNOS ratio was significantly reduced in 6J animals, highlighting an imbalance skewed toward proinflammatory macrophage activity in this substrain (Figure 4G).

These data underscore the pivotal role of B cells and macrophage polarization in modulating disease severity between both substrains, as 6J mice exhibit an immunologic milieu strongly influenced by effector B cells and macrophages with an M1 phenotype. On the contrary, the 6N substrain appears to maintain a less polarized B cell and macrophage response to the proinflammatory phenotype, which may partly explain its comparatively milder disease phenotype.

### 2.5 The Microbiome of both Substrains Shows Differences in Terms of Alpha and Beta Diversity

The microbiome is increasingly recognized as a key player in modulating immune responses and influencing the course of autoimmune diseases such as EAE. To explore potential baseline differences in the microbial community structure between the 6N and 6J substrains, both alpha‐ and beta‐diversity metrics were analyzed prior to EAE induction.

**FIGURE 5:**
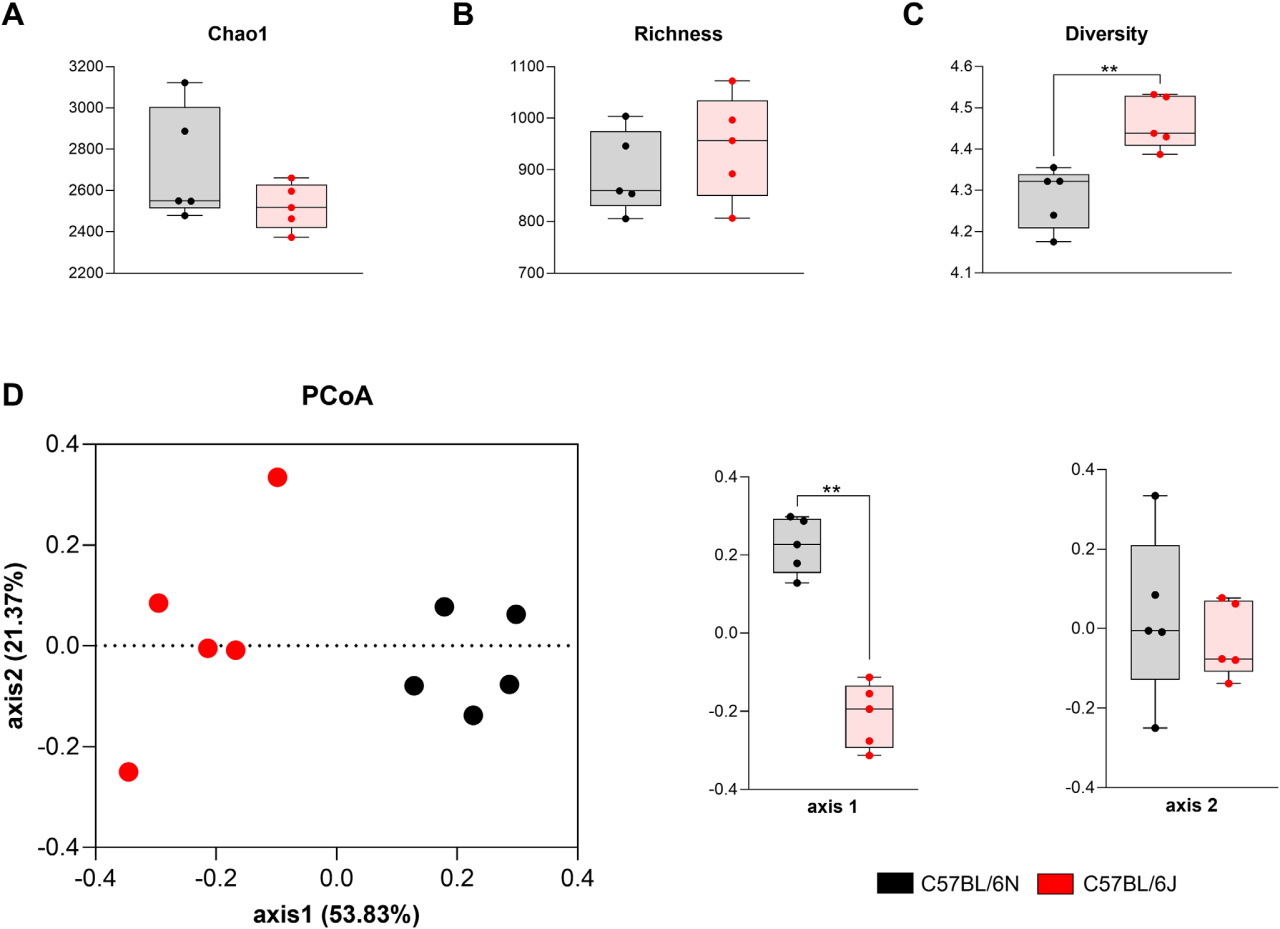
Alpha‐ and beta‐diversity of the intestinal microbiota in 6N and 6J mice. Chao1 (A), Richness (B), and Shannon index (C) were used to evaluate alpha diversity. PCoA plots (D) represent bacterial beta diversity based on the overall structure of the stool microbiota, calculated using Jaccard distances. Each dot represents an individual sample. Bars represent the mean ± SEM of C57BL/6N (*n* = 5) and C57BL/6J (*n* = 5) mice. *p* ≤ 0.01. PCoA, principal coordinate analysis.

Evenness, richness, andalpha diversity were assessed using Chao1, number of “species observed” (sobs), and the Shannon index, respectively. While Chao1 and richness did not reveal significant differences between substrains (Figure 5A,B), the Shannon index indicated significantly higher microbial diversity in 6J mice compared with 6N mice (Figure 5C). This suggests that, despite similar species richness, the proportion of different bacterial groups is more balanced and varied in the 6J substrain.

Beta diversity was evaluated to investigate differences in overall microbial composition between substrains. Principal coordinate analysis (PCoA), based on the Bray–Curtis dissimilarity matrix, revealed a clear separation of the microbial communities between 6N and 6J mice. This distinction was statistically confirmed by the analysis of molecular variance (AMOVA) test, indicating that the substrains harbor significantly different microbial environments (Figure 5D).

These findings highlight the existence of substrain‐specific microbiome signatures that could influence the observed differences in the immune responses and EAE severity once the mice are immunized.

### 2.6 Bacterial Species and Genus Associated with Multiple Sclerosis Are More Representative in the 6J Substrain Than in the 6N Substrain

To identify specific bacterial taxa that differ in abundance between 6N and 6J mice, we analyzed the microbial composition at various taxonomic levels.

**FIGURE 6:**
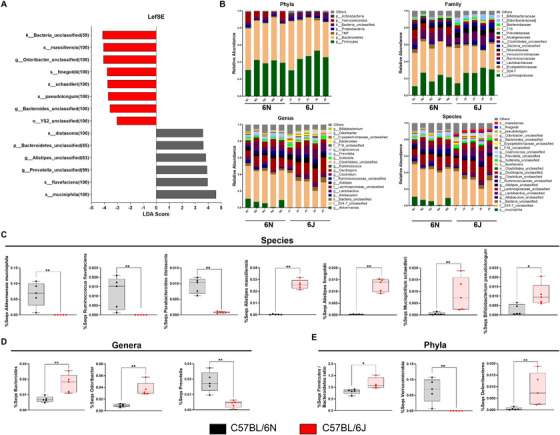
Compositional differences in fecal microbiota between 6J and 6N strains. Linear discriminant analysis (LDA) effect size (LEfSe) analysis (A) shows taxa with LDA > 3 and *p* < 0.05. Relative abundance at the phylum, family, genus, and species levels in 6J and 6N animals (B). Independent graphs of the relative abundance of specific microbial species including *Akkermansia muciniphila, Ruminoccocus flavefaciens, Parabacteroides distasonis, Alistipes massiliensis, Alistipes finegoldii, Mucispirillum schaedleri*, and *Bifidobacterium pseudolongum* (C); of specific microbial genera including *Bacteroides, Odoribacter*, and *Prevotella* (D); and specific microbial phyla including the *Firmicutes/Bacteroidetes* ratio, *Verrucomicrobia*, and *Deferribacteres* (E). Each dot represents an individual animal. Bars represent the mean ± SEM of C57BL/6N (*n* = 5) and C57BL/6J (*n* = 5) mice. *p ≤ 0.05; **p ≤ 0.01.

At the species level, significant differences were observed between substrains. While *Akkermansia municiphila*, *Ruminococcus flavefaciens*, and *Parabacteroides distasonis* were notably decreased in 6J animals, other species, such as *Bifidobacterium pseudolongum*, *Mucispirillum schaedleri*, and two species of the genus *Alistipes*—*Alistipes finegoldii* and *Alistipes massiliensis*—were significantly enriched in 6J compared with 6N mice (Figure 6A–C).

At the genus level, most genera associated with the aforementioned species followed similar abundance patterns, with the exception of the genus Ruminococcus (Figure 6B; Figure S1A). Additionally, three genera not related to any specific species—Bacteroides, Odoribacter, and Prevotella—also showed significant differences between substrains (Figure 6B,D).

The family‐level analysis mirrored these trends, as families associated with the analyzed genera followed the same patterns of abundance and significance (Figure 6B; Figure S1B).

At the phylum level, the Firmicutes:Bacteroidetes ratio, a marker often linked to immune and metabolic health, was significantly higher in 6J animals (Figure 6E). Differences were also evident in the relative abundance of the Verrucomicrobia, Deferribacteres, and Bacteroidetes phyla (Figure 6B,E; Figure S1C).

These findings indicate that the 6J substrain harbors a microbial composition more closely associated with multiple sclerosis, which could contribute to the observed differences in disease severity and immune responses in EAE mice.

## 3 Discussion

This study demonstrates for the first time that the C57BL/6J substrain has a more severe EAE than the C57BL/6N, with differences accompanied by an increase in the frequency and inflammatory profile of immune cells infiltrating the CNS. Furthermore, the differences observed in the basal microbial taxa between the two substrains are consistent with the differences observed at the immunological level. These findings highlight the possibility that therapeutic efficacy may vary depending on the substrain, as we have observed in C57BL/6J and C57BL/6N EAE animals treated with methylprednisolone (at 160 mg/kg; a dose equivalent that receives RR‐MS patients in relapse) or melatonin (Figures S2A–F). This finding underscores the critical need to characterize C57BL/6 substrains when evaluating novel therapies in preclinical models. By addressing this overlooked variability, the present work lays the groundwork for improving reproducibility and advancing precision in EAE research.

On the one hand, previous studies detected genomic variations between both substrains, including single nucleotide polymorphisms, small insertions and deletions, and structural variations [27]. On the other hand, Gharagozloo et al. observed that EAE Nlrp12 (−/−) mice, a gene affected in the 6J substrain, exhibited an exacerbated form of the disease compared with wild‐type mice [28], which points this genomic variation to the differential immune responses involved in the pathophysiology of EAE.

In the present work, a more severe disability is observed in the 6J substrain, with a significantly higher mean score at the peak of the disease, maximum, and cumulative score. Regarding the studies carried out in the EAE model that specified the substrain, 65 studies utilized the 6J substrain (Table S1) and only 7 used the 6N substrain (Table S2). Most of them were carried out in females and the most frequent ages were between 6 and 12 weeks old. To establish a reliable comparison of all studies in terms of disease severity, we adjusted their scoring criteria to our scoring system (described in Section 5). Despite the variability in sex, age, and immunization protocol used, the mean score at the peak of the disease in the 6J substrain was significantly higher than in the 6N substrain (Figure S2), which is consistent with our experimental results.

Since the severity of EAE correlates with the degree of leukocyte infiltration at CNS [29], the 6J animals presented an inflammatory infiltrate significantly greater than 6N. Although, the CD4^+^ T lymphocytes were equal between substrains, the populations of CD4^+^ T lymphocytes producing TNF, IFN‐γ, and IL‐17 were higher in 6J mice than in 6N mice. Furthermore, Treg and IL‐10 production by CD4^+^ T cells were higher in the 6N substrain than in 6J. These facts indicate a greater proinflammatory environment in the CNS of the 6J substrain compared with the 6N.

In addition, the 6J mice not only had a higher frequency of CD8^+^ T lymphocytes but also a more pronounced proinflammatory profile with a higher frequency of CD8^+^ T cells producing TNF, IFN‐γ, and IL‐17 in the 6J substrain. There is evidence that CD8^+^ T cells are present in lesions of MS patients, and correlate with disease progression [30]. These cells can induce oligodendrocyte death, transect axons, and promote vascular permeability that directly influences the neuropathology of EAE [31]. Therefore, the severity observed in the 6J substrain could be due in part to the action of CD8^+^ T cells in the CNS.

6J mice also showed a higher percentage of B cells in the CNS at the peak of disease, compared with the 6N. To our knowledge, this is the first study to show that the 6J substrain has a greater infiltration of TNF‐producing B lymphocytes compared with the 6N substrain. We also found that the frequency of B cells producing IL‐10 did not change between substrains, while the IL‐10/TNF ratio was higher in the 6N substrain. These results suggest that the profile of infiltrating B cells at the peak of the disease in 6N is more anti‐inflammatory than in the 6J substrain. Several lines of evidence also support the involvement of B cells in MS pathogenesis. Specifically, studies in human tissues from MS patients have shown B‐cell infiltration, particularly in tertiary lymphoid‐like structures in the meninges. Additionally, auto‐antibodies specific for myelin components were correlated with the degree of disease [32, 33]. Furthermore, MS patients exhibit abnormalities in B‐cell cytokine profiles. In particular, activated B cells from patients produce excessive amounts of TNF, among other proinflammatory cytokines [34]. Besides, IL‐10‐producing Breg cells are reduced during disease relapse, compared with remission [35]. To our knowledge, this is the first study to show that the 6J substrain has a greater CNS‐infiltration of TNF‐producing B lymphocytes compared with the 6N substrain. Therefore, the greater disability observed in the 6J substrain may be related to the increased presence of proinflammatory infiltrating B lymphocytes.

Although there are no previous studies comparing cytokine production between both substrains in EAE mice, literature widely describes that overproduction of proinflammatory cytokines is a hallmark of MS/EAE, and increased anti‐inflammatory cytokines contribute to resolving the disease [36, 37, 38]. Therefore, the increase of proinflammatory cytokines observed in the CNS of 6J is consistent with the greater severity of EAE observed in this substrain.

Here we report, for the first time, that 6J animals not only have a higher frequency of CNS‐infiltrating macrophages, but also these cells are more pathogenic than in the 6N substrain, as they exhibited higher production of TNF and iNOS, typical markers of the M1 profile. On the other hand, in the 6N substrain, the production of Arg1, a typical marker of the M2 profile, was more represented. It should be noted that the phenotypic switch of macrophages and microglia from M1 to M2 has received increasing attention as an approach to counteract the neuroinflammatory demyelination of MS, as evidenced by many studies [39, 40]. Indeed, recent findings highlighted the emerging role of M2 macrophages in controlling the progression of MS [41]. Based on these data, the differences observed in the macrophage population between both substrains could be also related to the the exacerbation of EAE in the 6J mice. In this line, macrophages are also required for the initiation of EAE [42] and the activation of microglia/macrophages correlates with the EAE progression [43].

Therefore, our results support the fact that the greater susceptibility to develop EAE in the 6J substrain could be related to the NLRP12 deficiency since it is a protein expressed mainly in myeloid cells and lymphocytes acting as a negative regulator of inflammation through the inhibition of NF‐κB [27, 44]. This would be especially related to the increased production of proinflammatory cytokines observed in both lymphocytes and macrophages in 6J mice, despite these being unstimulated cells.

The gut microbiome has been implicated in several autoimmune disorders, including rheumatoid arthritis, inflammatory bowel disease, and MS [17, 45]. Studies in EAE have shown that perturbation of the gut microbiota composition affects susceptibility to disease [46]. In the present study, the 6J mice have significantly higher alpha diversity, represented by the Shannon index, than the 6N substrain. These results are consistent with a previous study in which Shannon diversity was elevated in both RR‐MS and progressive MS compared with healthy controls [47]. With respect to beta diversity, the community composition was different between substrains, which was expected, since beta diversity has previously been detected to change between C57BL/6J and C57BL/6N substrains [48].

Regarding differences in the relative abundance of various microbial taxa between both substrains, we identified that the species *A. muciniphila*, its genus (*Akkermansia*), family (Verrucomicrobiaceae), and phylum (Verrucomicrobia), are significantly more abundant in 6N. Previously, Cox et al. found that *Akkermansia* isolated from MS patients improved EAE, which was related to a reduction in RORγt^+^ and IL‐17‐producing γδ T cells, suggesting a beneficial role for this genus in EAE and MS. Intestinal dysbiosis may also be influenced by a decrease in short‐chain fatty acid (SCFA)‐producing bacteria [49], including the Ruminococcaceae family [50, 51]. SCFAs are important immunoregulators with local and systemic effects [52]. They have been shown to mitigate the severity of EAE [53] by promoting Treg differentiation, FoxP3 expression, and blood‐brain barrier integrity [54, 55] while attenuating the frequency of Th1 cells [56]. Therefore, the decrease that we found in this study of *Ruminococcus flavefaciens* in the 6J substrain compared with the 6N could be related to the decrease in Treg cells and the increase in the Th1 response observed in the CNS of animals at the peak of the disease.

We also observed that two genera that comprise the Porphyromonadaceae family had opposite trends between 6N and 6J. While the genus *Odoribacter* increased in 6J, Parabacteroides and its species *P. distasonis* significantly decreased. In this line, a study showed increased *Odoribacter* in EAE mice compared with the healthy control [57]. This genus has been associated with proinflammatory processes and is also increased in a model of methamphetamine use disorder [58] and in patients with systemic lupus erythematosus [59]. Regarding *Parabacteroides*, a study carried out in MS patients showed that this genus was decreased with the disease. In fact, the authors created a disease prediction model in which *Parabacteroides* was included among the genera predictive of disease status [19]. Therefore, these data support that the 6J substrain has a microbiome composition that promotes the severity of EAE. Additionally, other families belonging to the Bacteroidetes phylum changed between 6N and 6J mice. In particular, the Prevotellaceae family and the *Prevotella* genus decreased in 6J, while the Rikenellaceae family, the *Alistipes* genus, and their species *A. massiliensis* and *A. finegoldii* increased. Many studies have indicated a decrease in *Prevotella* in MS patients [21, 60, 61] and a significant increase in the abundance after treatment with disease‐modifying therapy [17]. On the other hand, Reynders et al. showed an increase in the *Alistipes* genus in RR‐MS compared with the control [22], and the EAE score was correlated with significant increases in the relative abundance of *A. finegoldii* in ileal contents [23]. These results are also in agreement with the lower severity of the disease observed in the 6N substrain in the present study. Moreover, mice fed with a high‐fat diet (HFD) showed a higher abundance of *Odoribacter* and a lower abundance of the Prevotellaceae family. Furthermore, these animals had an elevated serum TNF level. On the contrary, mice fed an HFD and treated with stackyose (regulator of colonic and liver inflammation) decreased *Odoribacter* abundance and increased Prevotellaceae. Parallelly, the level of TNF was reduced [62]. Authors directly related these microbial taxa with the immune system. It is interesting that under HFD‐induced inflammation conditions, *Odoribacter* and Prevotellaceae showed the same tendency as the 6J substrain. These results point to the 6J substrain could have some basal systemic inflammation that makes it more susceptible to EAE. Moreover, a reduced level of *Prevotella* in patients with RR‐MS is associated with the expansion of Th17 cells and disease activity [17, 21]. Regarding *P. distasonis*, it is a low inducer of IFN‐γ and a very high inducer of IL‐10 in PBMC stimulated in vitro [63]. Thus, a microbiome with low levels of *P. distasonis* may have a more proinflammatory and less regulatory character, favoring the development of a more aggressive EAE, as occurs in 6J. Additionally, it is well known that a high‐salt diet promotes inflammatory effects through increased induction or activation of Th17 cells and M1‐like macrophages. Hamad et al. observed a significant enrichment in the relative abundance of *Alistipes* according to the sodium content of the food [64]. These findings may have important implications for inflammatory diseases caused by excessive salt intake since reducing this type of microbiota alleviates inflammatory parameters. We found an increase in the genera *A. massiliensis* and *A. finegoldii* in the microbiota of the 6J substrain, suggesting that the increase in Th17 and M1 cells could be related to a greater presence of the Alistipes genus.

Currently, there are no previous studies that related *M. schaedleri* to MS pathology. However, *M. schaedleri* can drive LPS production, which is associated with an inflammatory response, and its abundance has previously been correlated with the severity of intestinal inflammation [65]. Similarly, some studies have shown that *Mucispirillum* is associated with increased Th1 responses in the pathogenesis of colitis in mice [66, 67]. Our work reports that 6J animals had a significant increase in this bacterial species, which supports *M. schaedleri* as a possible pathological microbe that contributes to the increase in Th1 response involved in EAE. Otherwise, we are pioneers in describing differences in *Bifidobacterium pseudolongum* abundance between the 6J and 6N substrains. There are controversies in the literature on the role of the *Bifidobacterium* genus in MS. On the one hand, previous studies associated the lower frequency of *Bifidobacterium* in the intestinal tract with some risk factors for MS [68, 69]. On the other hand, increased levels of *Bifidobacterium* have been reported among MS patients ([50], [70]). Despite these variations, studies carried out in cancer mouse models revealed a connection between *B. pesudolongum* and an increase in IFN‐γ produced by CD4^+^ and CD8^+^ T cells [71]. Accordingly, we can consider that the increase in *B. pseudolongum* observed in the 6J substrain compared with 6N could trigger a greater response of effector CD4^+^ and CD8^+^ T lymphocytes, contributing to EAE development. We also observed that the genus *Bacteroides* is more present in the 6J microbiota. In a previous study, the authors detected a significantly higher production of antibacterial antibodies, including anti‐Bacteroides, in the CSF of subjects with demyelinating disease compared with healthy subjects. This supports the hypothesis that *Bacteroides*, among other microbes, contribute to demyelination in people with MS [72]. Furthermore, *Bacteroides fragilis* was shown to induce the maturation of the systemic Th1 response after colonization of germ‐free mice, through the production of unique types of polysaccharide A [73]. Based on these facts, the greater amount of Bacteroides observed in 6J in relation to 6N could lead to an increase in the Th1 response, leading to greater severity of EAE. Finally, we show, for the first time, a higher Firmicutes/Bacteroidetes ratio in 6J mice. There is evidence that the microbiota isolated from the small intestine of MS patients with high disease activity and a higher frequency of intestinal Th17 cells showed a higher Firmicutes/Bacteroidetes ratio compared with healthy controls and MS patients without disease activity [74]. Thus, an increase in this ratio in 6J mice could also predispose the animals to a more severe EAE.

In summary, the observed differences in EAE severity between the 6J and 6N substrains may be explained by a combination of genetic, immunological, and microbiota‐related factors. C57BL/6J mice carry a deletion in the *Nnt* gene, which affects mitochondrial function and antioxidant capacity, potentially exacerbating neuroinflammation and immune activation. Additionally, they harbor mutations in *Pde4b* and *Nlrp12*, both of which are involved in immune regulation and inflammatory signaling. *Pde4b* plays a crucial role in cyclic AMP signaling, which affects immune cell activation, while *Nlrp12* regulates inflammasome activity and immune homeostasis, potentially contributing to an increased inflammatory response in 6J mice. Immunologically, 6J mice exhibit a stronger proinflammatory T cell response (CD4^+^ and CD8^+^), whereas 6N mice show higher levels of anti‐inflammatory CD4^+^ cells in the CNS, which may help regulate disease severity. Furthermore, the basal microbiota composition significantly differs between substrains, with 6N mice showing a greater abundance of bacterial taxa associated with anti‐inflammatory and immunoregulatory effects, which can contribute to their reduced disease severity. These findings underscore the importance of substrain selection in EAE studies, as genetic background, immune responses, and microbiota composition collectively shape disease outcomes and may influence responses to therapeutic interventions.

Future studies could incorporate transcriptomic analysis to further unravel the molecular pathways underlying the differences observed between the 6J and 6N substrains. Such approaches would provide a more comprehensive understanding of the immunopathology of the disease, identifying novel targets relevant to EAE pathogenesis.

## 4 Conclusion

The present study shows for the first time that the C57BL/6J substrain has a more severe EAE than the C57BL/6N. The differences are accompanied by an increase in the frequency and inflammatory profile of the immune cells that infiltrate the CNS. Furthermore, the differences observed in basal microbial taxa between the two substrains could support the differences observed at the immunological level and therefore at the level of clinical signs and the development of EAE between the 6J and 6N substrains.

## 5 Materials and Methods

### 5.1 Animal Handling and Housing

Eight‐week‐old female C57BL/6N and C57BL/6J mice were purchased from the University of Seville Animal Facility. All experiments were approved by the Ethics Committee of the Virgen Macarena‐Virgen del Rocío University Hospital (reference numbers 24‐11‐15‐368 and 08/05/2024/055) and were carried out under Spanish legislation and the EU Directive 2010/63/EU for animal experiments. The mice were kept and housed under standard and pathogen‐free conditions (12/12 light/dark cycles, temperature 22 ± 2°C and humidity <55%) with ad libitum water and food at the Instituto de Biomedicina de Sevilla (IBiS).

### 5.2 EAE Induction and Clinical Evaluation

EAE was induced by subcutaneous immunization into the flanks with 100 µg of MOG_35–55_ (Cambridge Research Biochemicals) emulsified in CFA (Sigma) containing 50 µg of heat‐killed *Mycobacterium tuberculosis* (H37Ra, ATCC 25177) as previously described [9, 75, 76]. In addition, mice received two intraperitoneal injections of pertussis toxin (List Labs, California) at 200 ng per mouse on days 0 and 2 postinduction. The clinical signs of EAE were assessed daily as previously described [77].

### 5.3 Isolation of CNS Mononuclear Cells

Mice were sacrificed on day 15 postinduction, corresponding to the peak of the disease, and subjected to perfusion with ice‐cold sterile PBS. Brains and spinal cords were collected, minced, and enzymatically dissociated with 1.87 mg/mL of collagenase IV (Worthington) and 0.25 mg/mL of DNase I (AppliChem) for 35 min at 37°C to make a suspension of single cells. Mononuclear cells were then separated from the rest of the cells in 37%:70% discontinuous percoll gradients and were cultured at final densities of 2.5 × 10^6^ cells/mL with RPMI 1640 supplemented with 5% fetal bovine serum, 1% l‐glutamine, and 1% of penicillin/streptomycin. After overnight plated, cells were incubated with brefeldin A (eBioscience) for 5 h to study cytokine production and finally stained for analysis by flow cytometry.

### 5.4 Flow Cytometry

Mononuclear cells were stained for the following surface markers: CD45, CD4, CD25, CD8, CD19, CD11c, and CD11b, and dead cells were excluded using the LIVE/DEAD fixable dead cell stain kit (Invitrogen). After fixing and permeabilizing cells using FoxP3/transcription factor staining buffer set (eBioscience), they were stained intracellularly for the following intracellular markers: TNF, IFN‐γ, IL‐17, IL‐10, FoxP3, iNOS, and Arg1. See Table S3 for antibody characteristics. FACS analysis was performed using an LSR II Fortessa (BD) instrument, and data were analyzed using FlowJo software (Treestar). Representative gating strategies for all FACS experiments are shown in Figures S4–S6.

### 5.5 Microbiome Analysis

Stool samples were collected from C57BL/6N and C57BL/6J healthy mice, frozen at −80°C until DNA extraction. Fecal DNA was isolated using the E.Z.N.A. Stool DNA Kit (Omega BIO‐TEK) following the manufacturer's instructions and DNA quantity and quality were evaluated using the Qubit Fluorometer (Life Technologies). Subsequently, amplicon libraries of the V3‐ V4 region of the prokaryotic 16S rRNA gene were prepared according to the 16S metagenomics sequencing library preparation protocol (Part # 15044223 Rev. B). Paired‐end reads were sequenced on the MiSeq platform (Illumina).

Downstream analysis was performed in mothur. Sequences were quality‐filtered and aligned with the SILVA database. Then, chimeric sequences were discarded, and reads classified as mitochondria, cyanobacteria, eukaryote, or unknown were removed. Operational taxonomic units (OTUs) were clustered using a 97% similarity threshold and for taxonomic assignment, OTUs were compared with the Greengenes database v13.8.99 (http://greengenes.secondgenome.com).

Alpha diversity was calculated using the Shannon index, Chao1 method, and richness metrics. To assess the beta diversity between groups, the PCoA was used through the Jaccard index. Differential abundance analysis was performed using the LEfSE algorithm.

### 5.6 Statistics

Statistical analysis was performed using SPSS v24.0 software (IBM). The significance between groups was determined using the Mann–Whitney nonparametric *t*‐test. A *p‐*value ≤0.05 was considered statistically significant.

## Author Contributions

Antonio Carrillo‐Vico and Ana Isabel Álvarez‐López designed the study. Ana Isabel Álvarez‐López, Eduardo Ponce‐España, Ivan Cruz‐Chamorro, Guillermo Santos‐Sánchez, and Ignacio Bejarano performed experiments and analyzed experimental data. Eduardo Ponce‐España performed and analyzed the microbiota studies. Nuria Álvarez‐Sánchez, Patricia Judith Lardone, and Antonio Carrillo‐Vico supervision and funding acquisition. Antonio Carrillo‐Vico and Ana Isabel Álvarez‐López wrote the paper. All authors contributed to critically discussing the data and the manuscript. All authors approved the final version of the manuscript.

## Ethics Statement

All experiments were approved by the Ethics Committee of the Virgen Macarena‐Virgen del Rocío University Hospital (reference numbers: 24‐11‐15‐368 and 08/05/2024/055) and were carried out under Spanish legislation and the EU Directive 2010/63/EU for animal experiments.

## Conflicts of Interest

The authors declare no conflicts of interest.

## Supporting information



Supporting Information

## Data Availability

All data generated and analyzed during this study are available in the University of Seville research data repository (https://idus.us.es/handle/11441/32574). The raw 16S rRNA gene sequencing data have been deposited in the European Nucleotide Archive (ENA) under the accession number PRJEB88194 and will be publicly available upon publication at: https://www.ebi.ac.uk/ena/browser/view/PRJEB88194.
