# Supplementary material for: Differences of the 6N and 6J Substrains of C57BL/6 Mice in the Development of Experimental Autoimmune Encephalomyelitis
Source: MedComm (2020). 2025 Jul 2;6(7):e70228. doi: 10.1002/mco2.70228 (PMC12214946; doi:10.1002/mco2.70228)
Supplement: Supplementary file 1 — Supporting Information [file MCO2-6-e70228-s001.docx]

**Differences of the 6N and 6J substrains of C57BL/6 mice in the development of experimental autoimmune encephalomyelitis (EAE)**

Ana Isabel Álvarez-López^1,2^, Eduardo Ponce-España^1,2^, Ivan Cruz-Chamorro^1,2^, Guillermo Santos-Sánchez^1,2^, Ignacio Bejarano^1,2^, Nuria Álvarez-Sánchez^1^, Patricia Judith Lardone^1,2^, Antonio Carrillo-Vico^1,2,^*

Affiliations

^1^Instituto de Biomedicina de Sevilla, IBiS/Hospital Universitario Virgen del Rocío/CSIC/Universidad de Sevilla. Seville 41013, Spain.

^2^Departamento de Bioquímica Médica y Biología Molecular e Inmunología, Facultad de Medicina, Universidad de Sevilla. Seville 41009, Spain.

*Corresponding author.

Address correspondence and reprint requests to: Antonio Carrillo-Vico, Instituto de Biomedicina de Sevilla, IBIS/ Hospital Universitario Virgen del Rocío/CSIC/Universidad de Sevilla. Avda. Manuel Siurot s/n, 41013 Seville, Spain; Tel.: +34955923106; Fax: +34954907048; E-mail: [vico@us.es](mailto:vico@us.es)


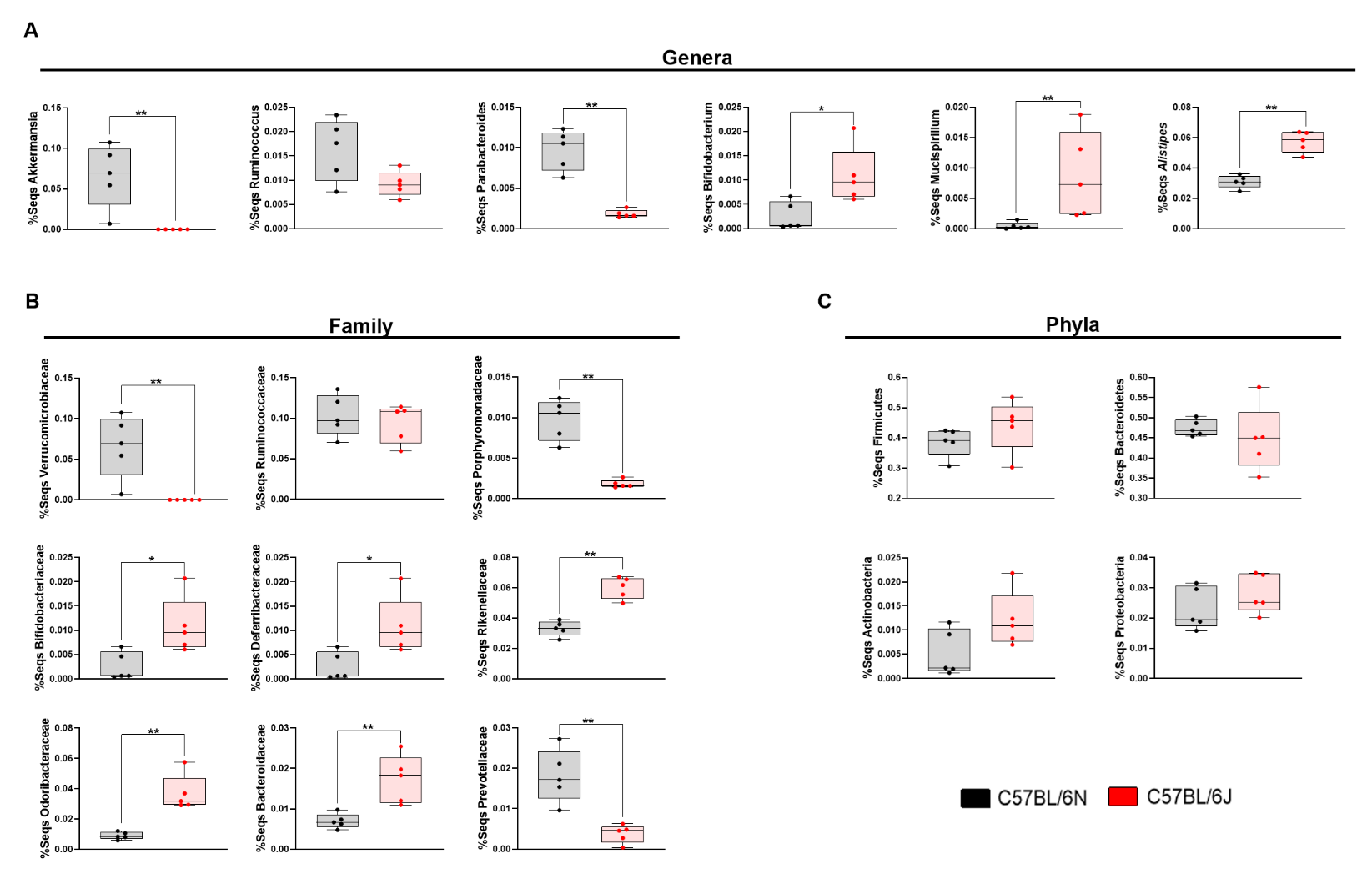


**Figure S1.** Differences in gut microbiota composition between 6J and 6N strain. Frequencies of genera: *Akkermansia, Ruminococcus, Parabacteroides, Bifidobacterium, Mucispirillum* and *Alistipes* (A). Frequencies of families: Verrucomicrobiaceae, Ruminococcaceae, Porphyromonodaceae, Bifidobacteriaceae, Deferribacteraceae, Rikenellaceae, Odoribacteraceae, Bacteroidaceae and Prevotellaceae (B). Frequencies of phyla: Firmicutes, Bacteroidetes, Actinobacteria and Proteobacteria (C). Each dot represents an individual sample. Bars represent the mean ± SEM of C57BL/6N (n = 5) and C57BL/6J (n = 5) mice. *p≤0.05; **p≤0.01.


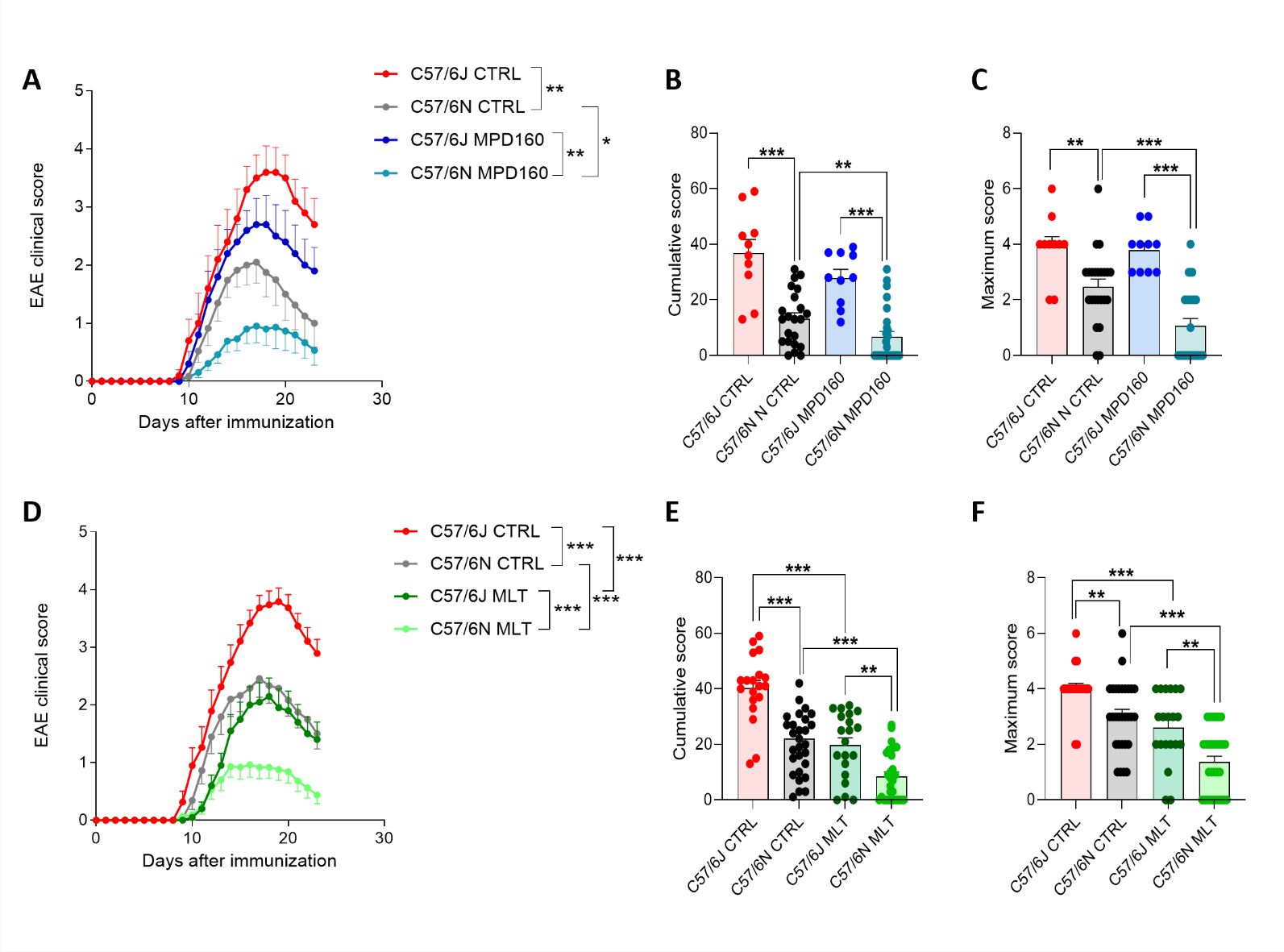
**Figure S2.** Effect of different treatments on the EAE development in C57BL/6J and C57BL/6N substrains. EAE clinical score curves (A), cumulative score (B) and maximum score (C) in C57BL/6J mice (n = 10) and C57BL/6N mice (n = 23) treated with vehicle (C57/6J CTRL and C57/6N CTRL, respectively); and in C57BL/6J mice (n= 10) and C57BL/6N mice (n= 26) treated with methylprednisolone at 160 mg/kg (C57/6J MPD160 and C57/6N MPD160, respectively). EAE clinical score curves (D), cumulative score (E) and maximum score (F) in C57BL/6J mice (n = 19) and C57BL/6N mice (n = 29) treated with vehicle (C57/6J CTRL and C57/6N CTRL, respectively); and in C57BL/6J mice (n = 20) and C57BL/6N mice (n = 30) treated with melatonin (C57/6J MLT and C57/6N MLT, respectively). Statistical analysis was performed using the Mann Whitney non-parametric t test. Each dot represents an individual animal. Bars represent the mean ± SEM. *p≤0.05; **p≤0.01; **p≤0.001.


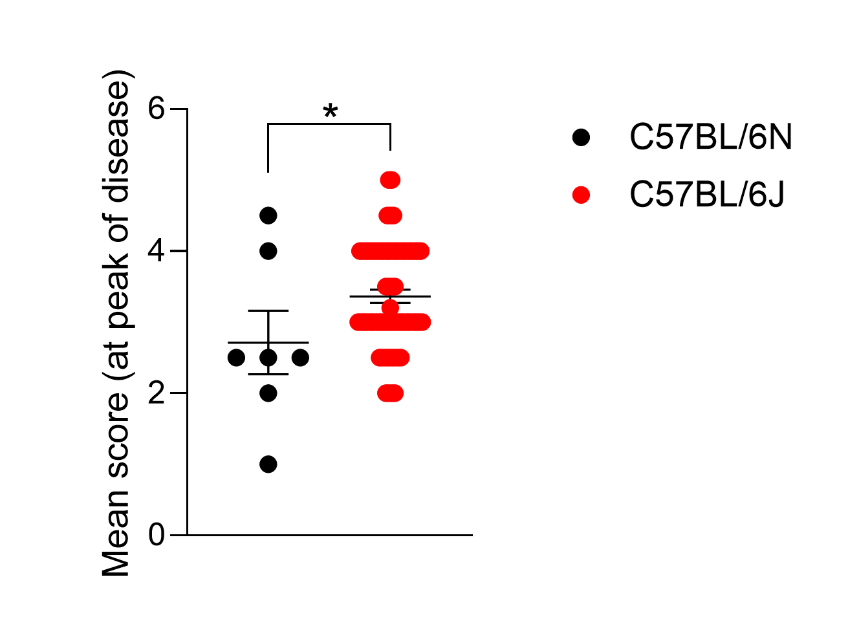


**Figure S3.** Differences in the severity of EAE between 6N and 6J strains based on data extracted from the literature. Mean clinical scores at the peak of disease in C57BL/6N and C57BL/6J mice were compiled from different studies identified in the bibliography. Each dot represents an individual study. Bars represent the mean ± SEM.


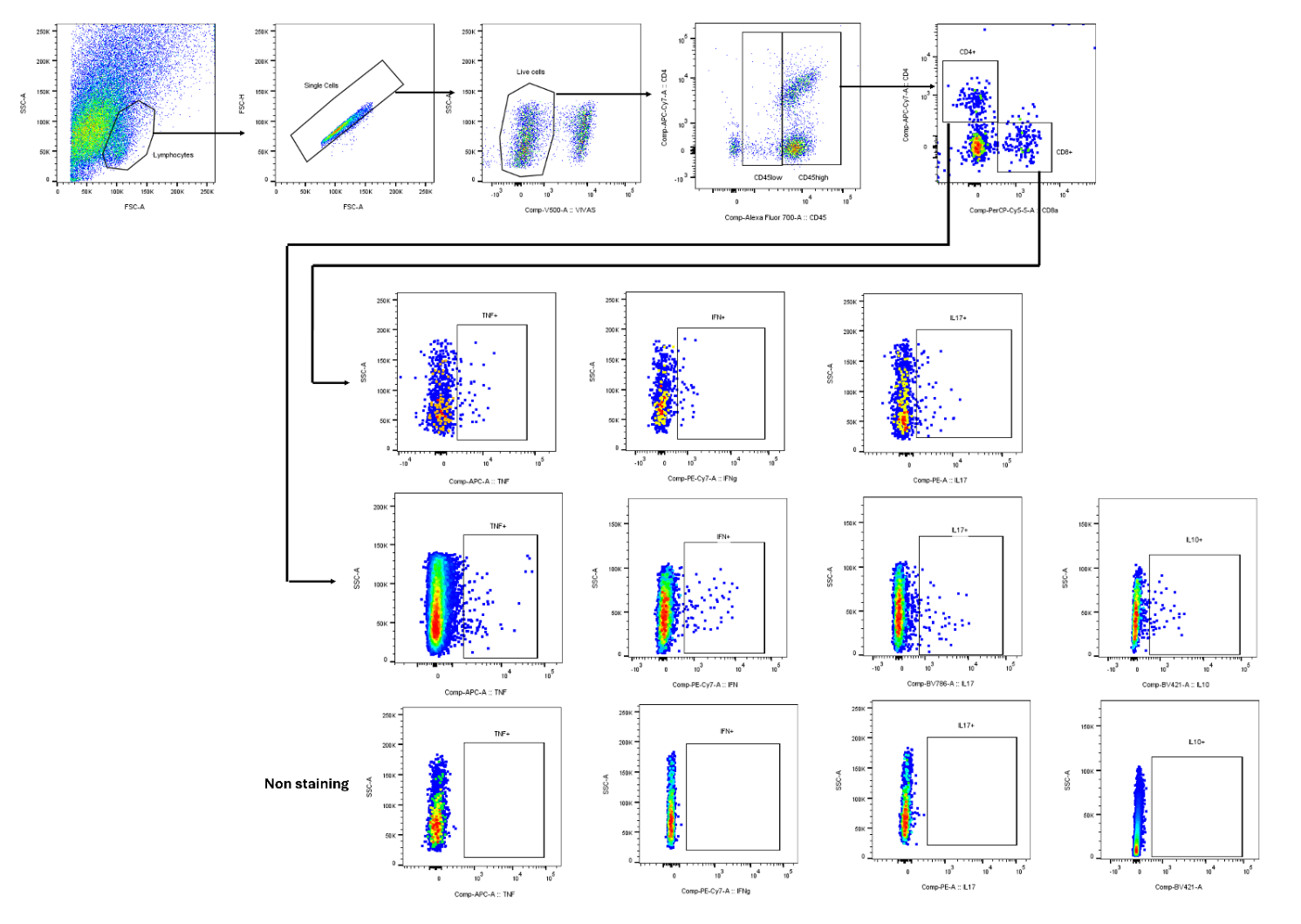


**Figure S4.** Representative gating strategies used to analyze flow cytometry data from CD4^+^ and CD8^+^ cells.


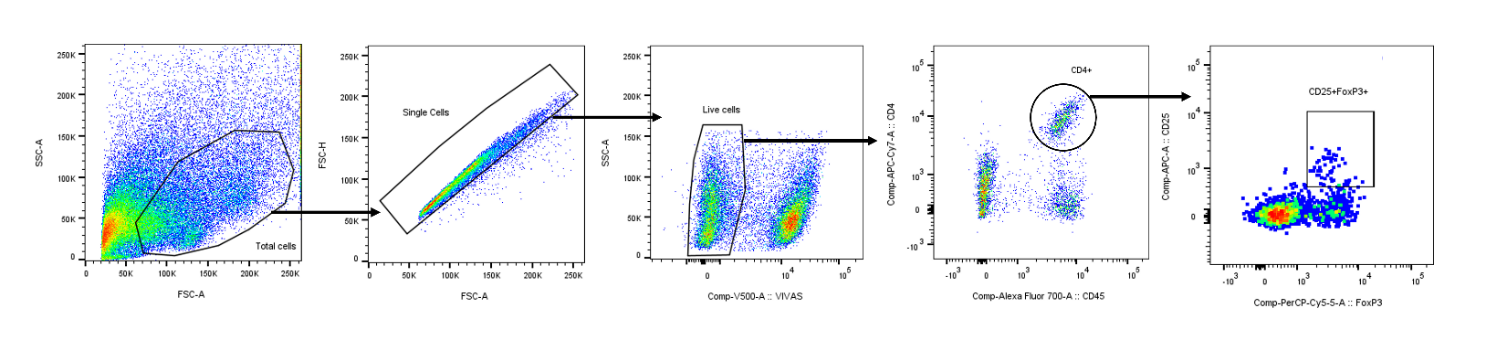
**Figure S5.** Representative gating strategies used to analyze flow cytometry data from regulatory T cells subset.


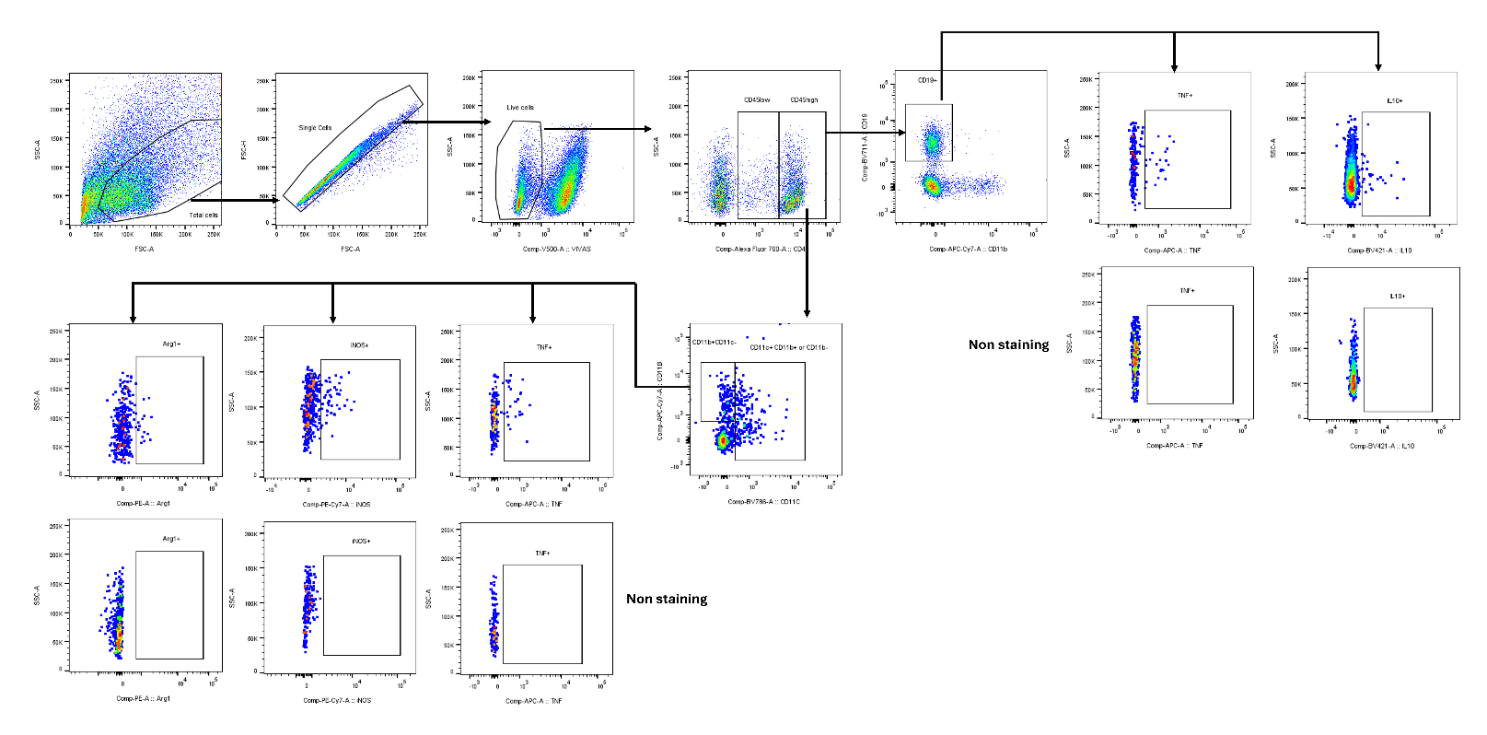


**Figure S6.** Representative gating strategies used to analyze flow cytometry data from macrophages, dendritic cells and B cells populations.

**Table S1.** Studies carried out in 6J substrain.

| **Substrain** | **Sex** | **Age (weeks)** | **Medium Score (at peak of disease)** | **Study** |
| --- | --- | --- | --- | --- |
| 6J | female | 6 | 4 | (1) |
| 6J | female | 8-9 | 3 | (2) |
| 6J | female | 10 | 2,5 | (3) |
| 6J | female | 8-10 | 4 | (4) |
| 6J | female | 16 | 3,5 | (5) |
| 6J | male | 8-9 | 2,5 | (6) |
| 6J | female | 4-6 | 4 | (7) |
| 6J | male and female | 8-10 | 4,5 | (8) |
| 6J | male and female | 8-12 | 4 | (9) |
| 6J | female | 7-8 | 3 | (10) |
| 6J | female | 4-6 | 2,5 | (11) |
| 6J | female | 6-8 | 2 | (12) |
| 6J | female | 9-13 | 2 | (13) |
| 6J | female | 8-12 | 4,5 | (14) |
| 6J | female | 6 | 3 | (15) |
| 6J | female | 6 | 3 | (16) |
| 6J | female | 8-12 | 3 | (17) |
| 6J | female | 6-8 | 3 | (18) |
| 6J | female | 6-7 | 3,2 | (19) |
| 6J | female | 8-12 | 4 | (20) |
| 6J | male and female | 9-10 | 3 | (21) |
| 6J | female | 11-12 | 4 | (22) |
| 6J | male | 8-12 | 3 | (23) |
| 6J | female | 9 | 3 | (24) |
| 6J | female | 8-10 | 4 | (25) |
| 6J | female | 9-11 | 3,5 | (26) |
| 6J | male and female | 7 | 3 | (27) |
| 6J | male and female | 8-10 | 2,5 | (28) |
| 6J | female | 12-15 | 2,5 | (29) |
| 6J | female | 14 | 4 | (30) |
| 6J | female | 8-12 | 5 | (31) |
| 6J | female | 9-10 | 3 | (32) |
| 6J | female | 7-8 | 2 | (33) |
| 6J | female | 4-6 | 3 | (34) |
| 6J | female | 8-12 | 3 | (35) |
| 6J | female | 16-18 | 4 | (36) |
| 6J | female | 6 | 2,5 | (37) |
| 6J | - | 8 | 2,5 | (38) |
| 6J | female | 8 | 3,5 | (39) |
| 6J | female | 10 | 3 | (40) |
| 6J | male and female | 6-22 | 3 | (41) |
| 6J | female | 6 | 3 | (42) |
| 6J | female | 6-8 | 4,5 | (43) |
| 6J | female | 6-8 | 3 | (44) |
| 6J | female | 6-8 | 4 | (45) |
| 6J | female | 11-13 | 4 | (46) |
| 6J | female | 8-12 | 4 | (47) |
| 6J | female | 10 | 4 | (48) |
| 6J | female | 5 | 3 | (49) |
| 6J | female | 6-8 | 4 | (50) |
| 6J | female | 8 | 4 | (51) |
| 6J | female | 6-8 | 3 | (52) |
| 6J | male and female | 8 | 4 | (53) |
| 6J | female | 6 | 4 | (54) |
| 6J | female | 8 | 4 | (55) |
| 6J | female | 7 | 4 | (56) |
| 6J | female | - | 5 | (57) |
| 6J | - | 8 | 3,5 | (58) |
| 6J | female | 6-12 | 3 | (59) |
| 6J | male and female | - | 4 | (60) |
| 6J | female | 6-8 | 4 | (61) |
| 6J | female | 8-12 | 2,5 | (62) |
| 6J | female | 6-8 | 3 | (63) |
| 6J | female | 6-8 | 3 | (64) |
| 6J | female | 4 | 2 | (65) |

**Table S2.** Studies carried out in 6N substrain.

| **Substrain** | **Sex** | **Age (weeks)** | **Medium Score (at peak of disease)** | **Study** |
| --- | --- | --- | --- | --- |
| 6N | female | 8 | 2,5 | (66) |
| 6N | male and female | 8 | 1 | (67) |
| 6N | female | 6-8 | 2 | (68) |
| 6N | female | 10-12 | 4 | (69) |
| 6N | female | 10-12 | 4,5 | (70) |
| 6N | female | 10-12 | 2,5 | (71) |
| 6N | female | 6-8 | 2,5 | (72) |

**Table S3.** Characteristics of the antibodies used for flow cytometry in this study.

| **Antibody** | **Fluorochrome** | **Clone** | **Manufacturer** |
| --- | --- | --- | --- |
| Rat antimouse CD45 | AF700 | 30-F11 | BD Biosciences |
| Rat antimouse CD4 | APC-Cy7 | GK1.5 | BD Biosciences |
| Rat antimouse CD8α | PerCP-Cy5.5 | 53-6.7 | BD Biosciences |
| Rat antimouse CD19 | BV711 | 1D3 | BD Biosciences |
| Rat anti-mouse CD11b | APC-Cy7 | M1/70 | BD Biosciences |
| Hamster anti-mouse CD11c | BV786 | HL3 | BD Biosciences |
| Rat anti-mouse iNOS | PE-Cy7 | CXNFT | eBioscience |
| Sheep anti-mouse ARG1 | PE | Polyclonal | R&D |
| Rat anti-mouse IL-10 | BV421 | JES5-16E3 | BD Biosciences |
| Rat anti-mouse IFNγ | PE-Cy7 | XMG1.2 | BD Biosciences |
| Rat anti-mouse TNF | APC | MP6-XT22 | BD Biosciences |
| Rat anti-mouse IL-17A | PE | TC11-18H10 | BD Biosciences |
| Rat anti-mouse CD25 | APC | 3C7 | BD Biosciences |
| Rat anti-mouse FoxP3 | PerCP-Cy5.5 | R16-715 | BD Biosciences |

**References**

1. Mabrouk M, El Ayed M, Démosthènes A, Aissouni Y, Aouani E, Daulhac-Terrail L, et al. Antioxidant effect of grape seed extract corrects experimental autoimmune encephalomyelitis behavioral dysfunctions, demyelination, and glial activation. Frontiers in Immunology. 2022.

2. Hoelz AG, Bernardes D, Cartarozzi LP, de Oliveira ALR. Gliosis attenuation in experimental autoimmune encephalomyelitis by a combination of dimethyl fumarate and pregabalin. Frontiers in Cellular Neuroscience. 2022;16.

3. Titus HE, Xu H, Robinson AP, Patel PA, Chen Y, Fantini D, et al. Repurposing the cardiac glycoside digoxin to stimulate myelin regeneration in chemically‐induced and immune‐mediated mouse models of multiple sclerosis. Glia. 2022;70(10):1950-70.

4. Chen Z, Fan R, Liang J, Xiao Z, Dang J, Zhao J, et al. NFIL3 deficiency alleviates EAE through regulating different immune cell subsets. Journal of advanced research. 2022;39:225-35.

5. Shi C, Cha J, Gong J, Wang S, Zeng P, Lian J, et al. Amelioration of Experimental Autoimmune Encephalomyelitis in Alzheimer’s Disease Mouse Models: A Potential Role for Aβ. Cells. 2022;11(6):1004.

6. Joly S, Mdzomba JB, Rodriguez L, Morin F, Vallières L, Pernet V. B cell-dependent EAE induces visual deficits in the mouse with similarities to human autoimmune demyelinating diseases. Journal of neuroinflammation. 2022;19(1):1-17.

7. Lang Y, Chu F, Liu L, Zheng C, Li C, Shen D, et al. Potential role of BAY11-7082, a NF-κB blocker inhibiting experimental autoimmune encephalomyelitis in C57BL/6J mice via declining NLRP3 inflammasomes. Clinical and experimental immunology. 2022;207(3):378-86.

8. Hao W, Luo Q, Menger MD, Fassbender K, Liu Y. Treatment with CD52 antibody protects neurons in experimental autoimmune encephalomyelitis mice during the recovery phase. Frontiers in immunology. 2021:5475.

9. Huntemann N, Vogelsang A, Groeneweg L, Willison A, Herrmann AM, Meuth SG, Eichler S. An optimized and validated protocol for inducing chronic experimental autoimmune encephalomyelitis in C57BL/6J mice. Journal of Neuroscience Methods. 2022;367:109443.

10. Pinto BF, Ribeiro LNB, Da Silva GBRF, Freitas CS, Kraemer L, Oliveira FMS, et al. Inhalation of dimethyl fumarate-encapsulated solid lipid nanoparticles attenuate clinical signs of experimental autoimmune encephalomyelitis and pulmonary inflammatory dysfunction in mice. Clinical Science. 2022;136(1):81-101.

11. Dalenogare DP, Theisen MC, Peres DS, Fialho MF, Andrighetto N, Barros L, et al. Transient receptor potential ankyrin 1 mediates headache-related cephalic allodynia in a mouse model of relapsing–remitting multiple sclerosis. Pain. 2022;163(7):1346-55.

12. Errede M, Annese T, Petrosino V, Longo G, Girolamo F, de Trizio I, et al. Microglia-derived CCL2 has a prime role in neocortex neuroinflammation. Fluids and Barriers of the CNS. 2022;19(1):1-25.

13. Smith KJ, Minns D, McHugh BJ, Holloway RK, O’Connor R, Williams A, et al. The antimicrobial peptide cathelicidin drives development of experimental autoimmune encephalomyelitis in mice by affecting Th17 differentiation. PLoS biology. 2022;20(8):e3001554.

14. Vandenbark AA, Meza-Romero R, Wiedrick J, Gerstner G, Seifert H, Kent G, et al. “Near Cure” treatment of severe acute EAE in MIF-1-deficient female and male mice with a bifunctional MHCII-derived molecular construct. Cellular Immunology. 2022;378:104561.

15. Deak P, Knight HR, Esser-Kahn A. Robust tolerogenic dendritic cells via push/pull pairing of toll-like-receptor agonists and immunomodulators reduces EAE. Biomaterials. 2022;286:121571.

16. Cossu D, Yokoyama K, Sakanishi T, Kuwahara-Arai K, Momotani E, Hattori N. A mucosal immune response induced by oral administration of heat-killed Mycobacterium avium subsp. paratuberculosis exacerbates EAE. Journal of Neuroimmunology. 2021;352:577477.

17. Rodero M, Cuéllar C. Modulation by Anisakis simplex antigen of inflammatory response generated in experimental autoimmune encephalomyelitis. International Immunopharmacology. 2021;90:107241.

18. Baniahmad A, Birkner K, Görg J, Loos J, Zipp F, Wasser B, Bittner S. The frequency of follicular T helper cells differs in acute and chronic neuroinflammation. Scientific reports. 2020;10(1):1-11.

19. Patel C, Meadowcroft MD, Zagon IS, McLaughlin PJ. [Met5]-enkephalin preserves diffusion metrics in EAE mice. Brain Research Bulletin. 2020;165:246-52.

20. Hoghooghi V, Palmer AL, Frederick A, Jiang Y, Merkens JE, Balakrishnan A, et al. Cystatin C plays a sex-dependent detrimental role in experimental autoimmune encephalomyelitis. Cell reports. 2020;33(1):108236.

21. Sanchez JMS, Doty DJ, DePaula-Silva AB, Brown DG, Bell R, Klag KA, et al. Molecular patterns from a human gut-derived Lactobacillus strain suppress pathogenic infiltration of leukocytes into the central nervous system. Journal of neuroinflammation. 2020;17(1):1-14.

22. Stevens MY, Cropper HC, Lucot KL, Chaney AM, Lechtenberg KJ, Jackson IM, et al. Development of a CD19 PET tracer for detecting B cells in a mouse model of multiple sclerosis. Journal of neuroinflammation. 2020;17(1):1-11.

23. Wetzel LA, Hurtado M, Kaswan ZAM, McCusker RH, Steelman AJ. Deletion of indoleamine 2, 3 dioxygenase (Ido) 1 but not Ido2 exacerbates disease symptoms of MOG35-55-induced experimental autoimmune encephalomyelitis. Brain, Behavior, & Immunity-Health. 2020;7:100116.

24. De Oliveira LRC, Mimura LAN, Fraga-Silva TFDC, Ishikawa LLW, Fernandes AAH, Zorzella-Pezavento SFG, Sartori A. Calcitriol prevents neuroinflammation and reduces blood-brain barrier disruption and local macrophage/microglia activation. Frontiers in Pharmacology. 2020;11:161.

25. Heimann AS, Giardini AC, Sant’Anna M, Dos Santos NB, Gewehr MC, Munhoz CD, et al. NFKF is a synthetic fragment derived from rat hemopressin that protects mice from neurodegeneration. Neuroscience Letters. 2020;721:134765.

26. Thiele née Schrewe L, Guse K, Tietz S, Remlinger J, Demir S, Pedreiturria X, et al. Functional relevance of the multi-drug transporter abcg2 on teriflunomide therapy in an animal model of multiple sclerosis. Journal of neuroinflammation. 2020;17(1):1-9.

27. Caslin B, Maguire C, Karmakar A, Mohler K, Wylie D, Melamed E. Alcohol shifts gut microbial networks and ameliorates a murine model of neuroinflammation in a sex-specific pattern. Proceedings of the National Academy of Sciences. 2019;116(51):25808-15.

28. Cruz-Herranz A, Dietrich M, Hilla AM, Yiu HH, Levin MH, Hecker C, et al. Monitoring retinal changes with optical coherence tomography predicts neuronal loss in experimental autoimmune encephalomyelitis. Journal of neuroinflammation. 2019;16(1):1-17.

29. Migotto M-A, Mardon K, Orian J, Weckbecker G, Kneuer R, Bhalla R, Reutens DC. Efficient Distribution of a Novel Zirconium-89 Labeled Anti-cd20 Antibody Following Subcutaneous and Intravenous Administration in Control and Experimental Autoimmune Encephalomyelitis-Variant Mice. Frontiers in immunology. 2019;10:2437.

30. Zyla K, Larabee CM, Georgescu C, Berkley C, Reyna T, Plafker SM. Dimethyl fumarate mitigates optic neuritis. Molecular vision. 2019;25:446.

31. Tsai H-C, Nguyen K, Hashemi E, Engleman E, Hla T, Han MH. Myeloid sphingosine-1-phosphate receptor 1 is important for CNS autoimmunity and neuroinflammation. Journal of Autoimmunity. 2019;105:102290.

32. Cossu D, Yokoyama K, Sakanishi T, Momotani E, Hattori N. Adjuvant and antigenic properties of Mycobacterium avium subsp. paratuberculosis on experimental autoimmune encephalomyelitis. Journal of neuroimmunology. 2019;330:174-7.

33. Pol S, Schweser F, Bertolino N, Preda M, Sveinsson M, Sudyn M, et al. Characterization of leptomeningeal inflammation in rodent experimental autoimmune encephalomyelitis (EAE) model of multiple sclerosis. Experimental Neurology. 2019;314:82-90.

34. Bernardes D, de Oliveira ALR. Regular Exercise Modifies Histopathological Outcomes of Pharmacological Treatment in Experimental Autoimmune Encephalomyelitis. Front Neurol. 2018;9:950.

35. Tietz S, Périnat T, Greene G, Enzmann G, Deutsch U, Adams R, et al. Lack of junctional adhesion molecule (JAM)-B ameliorates experimental autoimmune encephalomyelitis. Brain Behav Immun. 2018;73:3-20.

36. Manogaran P, Walker-Egger C, Samardzija M, Waschkies C, Grimm C, Rudin M, Schippling S. Exploring experimental autoimmune optic neuritis using multimodal imaging. Neuroimage. 2018;175:327-39.

37. Dietrich M, Helling N, Hilla A, Heskamp A, Issberner A, Hildebrandt T, et al. Early alpha-lipoic acid therapy protects from degeneration of the inner retinal layers and vision loss in an experimental autoimmune encephalomyelitis-optic neuritis model. J Neuroinflammation. 2018;15(1):71.

38. McDougald DS, Dine KE, Zezulin AU, Bennett J, Shindler KS. SIRT1 and NRF2 Gene Transfer Mediate Distinct Neuroprotective Effects Upon Retinal Ganglion Cell Survival and Function in Experimental Optic Neuritis. Invest Ophthalmol Vis Sci. 2018;59(3):1212-20.

39. González-García C, Torres IM, García-Hernández R, Campos-Ruíz L, Esparragoza LR, Coronado MJ, et al. Mechanisms of action of cannabidiol in adoptively transferred experimental autoimmune encephalomyelitis. Exp Neurol. 2017;298(Pt A):57-67.

40. Aarts S, Seijkens TTP, Kusters PJH, van der Pol SMA, Zarzycka B, Heijnen P, et al. Inhibition of CD40-TRAF6 interactions by the small molecule inhibitor 6877002 reduces neuroinflammation. J Neuroinflammation. 2017;14(1):105.

41. Reynolds JD, Case LK, Krementsov DN, Raza A, Bartiss R, Teuscher C. Modeling month-season of birth as a risk factor in mouse models of chronic disease: from multiple sclerosis to autoimmune encephalomyelitis. Faseb j. 2017;31(6):2709-19.

42. Martín-Álvarez R, Paúl-Fernández N, Palomo V, Gil C, Martínez A, Mengod G. A preliminary investigation of phoshodiesterase 7 inhibitor VP3.15 as therapeutic agent for the treatment of experimental autoimmune encephalomyelitis mice. J Chem Neuroanat. 2017;80:27-36.

43. Ozgun-Acar O, Celik-Turgut G, Gazioglu I, Kolak U, Ozbal S, Ergur BU, et al. Capparis ovata treatment suppresses inflammatory cytokine expression and ameliorates experimental allergic encephalomyelitis model of multiple sclerosis in C57BL/6 mice. J Neuroimmunol. 2016;298:106-16.

44. McLaughlin PJ, McHugh DP, Magister MJ, Zagon IS. Endogenous opioid inhibition of proliferation of T and B cell subpopulations in response to immunization for experimental autoimmune encephalomyelitis. BMC Immunol. 2015;16:24.

45. Guo X, Namekata K, Kimura A, Noro T, Azuchi Y, Semba K, et al. Brimonidine suppresses loss of retinal neurons and visual function in a murine model of optic neuritis. Neurosci Lett. 2015;592:27-31.

46. Franken J, Gevaert T, Uvin P, Wauterickx K, Boeve AC, Rietjens R, et al. Urodynamic changes in mice with experimental autoimmune encephalomyelitis correlate with neurological impairment. Neurourol Urodyn. 2016;35(4):450-6.

47. Abadier M, Haghayegh Jahromi N, Cardoso Alves L, Boscacci R, Vestweber D, Barnum S, et al. Cell surface levels of endothelial ICAM-1 influence the transcellular or paracellular T-cell diapedesis across the blood-brain barrier. Eur J Immunol. 2015;45(4):1043-58.

48. Pryor WM, Freeman KG, Larson RD, Edwards GL, White LJ. Chronic exercise confers neuroprotection in experimental autoimmune encephalomyelitis. J Neurosci Res. 2015;93(5):697-706.

49. Sands SA, Tsau S, Yankee TM, Parker BL, Ericsson AC, LeVine SM. The effect of omeprazole on the development of experimental autoimmune encephalomyelitis in C57BL/6J and SJL/J mice. BMC Res Notes. 2014;7:605.

50. Kuerten S, Wunsch M, Lehmann PV. Longitudinal T cell-derived IFN-γ/IL-17 balances do not correlate with the disease course in two mouse models of experimental autoimmune encephalomyelitis. J Immunol Methods. 2013;398-399:68-75.

51. Achiron A, Mashiach R, Zilkha-Falb R, Meijler MM, Gurevich M. Polymerase I pathway inhibitor ameliorates experimental autoimmune encephalomyelitis. J Neuroimmunol. 2013;263(1-2):91-7.

52. Semon JA, Zhang X, Pandey AC, Alandete SM, Maness C, Zhang S, et al. Administration of murine stromal vascular fraction ameliorates chronic experimental autoimmune encephalomyelitis. Stem Cells Transl Med. 2013;2(10):789-96.

53. Krementsov DN, Katchy A, Case LK, Carr FE, Davis B, Williams C, Teuscher C. Studies in experimental autoimmune encephalomyelitis do not support developmental bisphenol a exposure as an environmental factor in increasing multiple sclerosis risk. Toxicol Sci. 2013;135(1):91-102.

54. Bowie LE, Roscoe WA, Lui EM, Smith R, Karlik SJ. Effects of an aqueous extract of North American ginseng on MOG(35-55)-induced EAE in mice. Can J Physiol Pharmacol. 2012;90(7):933-9.

55. Yoshida Y, Tsuji T, Fujita T, Kohno T. Relapse of experimental autoimmune encephalomyelitis after discontinuation of FTY720 (Fingolimod) treatment, but not after combination of FTY720 and pathogenic autoantigen. Biol Pharm Bull. 2011;34(6):933-6.

56. Freria CM, Zanon RG, Santos LM, Oliveira AL. Major histocompatibility complex class I expression and glial reaction influence spinal motoneuron synaptic plasticity during the course of experimental autoimmune encephalomyelitis. J Comp Neurol. 2010;518(7):990-1007.

57. Fujita M, Otsuka T, Mizuno M, Tomi C, Yamamura T, Miyake S. Carcinoembryonic antigen-related cell adhesion molecule 1 modulates experimental autoimmune encephalomyelitis via an iNKT cell-dependent mechanism. Am J Pathol. 2009;175(3):1116-23.

58. Osmers I, Smith SS, Parks BW, Yu S, Srivastava R, Wohler JE, et al. Deletion of the G2A receptor fails to attenuate experimental autoimmune encephalomyelitis. J Neuroimmunol. 2009;207(1-2):18-23.

59. Brown DA, Sawchenko PE. Time course and distribution of inflammatory and neurodegenerative events suggest structural bases for the pathogenesis of experimental autoimmune encephalomyelitis. J Comp Neurol. 2007;502(2):236-60.

60. Teuscher C, Noubade R, Spach K, McElvany B, Bunn JY, Fillmore PD, et al. Evidence that the Y chromosome influences autoimmune disease in male and female mice. Proc Natl Acad Sci U S A. 2006;103(21):8024-9.

61. Zappia E, Casazza S, Pedemonte E, Benvenuto F, Bonanni I, Gerdoni E, et al. Mesenchymal stem cells ameliorate experimental autoimmune encephalomyelitis inducing T-cell anergy. Blood. 2005;106(5):1755-61.

62. Petersen TR, Bettelli E, Sidney J, Sette A, Kuchroo V, Bäckström BT. Characterization of MHC- and TCR-binding residues of the myelin oligodendrocyte glycoprotein 38-51 peptide. Eur J Immunol. 2004;34(1):165-73.

63. Weir C, Bernard CC, Bäckström BT. IL-5-deficient mice are susceptible to experimental autoimmune encephalomyelitis. Int Immunol. 2003;15(11):1283-9.

64. Okuda Y, Okuda M, Bernard CC. Regulatory role of p53 in experimental autoimmune encephalomyelitis. J Neuroimmunol. 2003;135(1-2):29-37.

65. Sanna V, Di Giacomo A, La Cava A, Lechler RI, Fontana S, Zappacosta S, Matarese G. Leptin surge precedes onset of autoimmune encephalomyelitis and correlates with development of pathogenic T cell responses. J Clin Invest. 2003;111(2):241-50.

66. Álvarez-Sánchez N, Cruz-Chamorro I, Álvarez-López AI, López-González A, Lacalle Remigio JR, Lardone PJ, et al. Seasonal Variations in Macrophages/Microglia Underlie Changes in the Mouse Model of Multiple Sclerosis Severity. Mol Neurobiol. 2020;57(10):4082-9.

67. Mirabelli E, Ni L, Li L, Acioglu C, Heary RF, Elkabes S. Pathological pain processing in mouse models of multiple sclerosis and spinal cord injury: contribution of plasma membrane calcium ATPase 2 (PMCA2). J Neuroinflammation. 2019;16(1):207.

68. Papaneophytou CP, Georgiou E, Karaiskos C, Sargiannidou I, Markoullis K, Freidin MM, et al. Regulatory role of oligodendrocyte gap junctions in inflammatory demyelination. Glia. 2018;66(12):2589-603.

69. Blum L, Tafferner N, Spring I, Kurz J, deBruin N, Geisslinger G, et al. Dietary phytol reduces clinical symptoms in experimental autoimmune encephalomyelitis (EAE) at least partially by modulating NOX2 expression. J Mol Med (Berl). 2018;96(10):1131-44.

70. Bianchi B, Smith PA, Abriel H. The ion channel TRPM4 in murine experimental autoimmune encephalomyelitis and in a model of glutamate-induced neuronal degeneration. Mol Brain. 2018;11(1):41.

71. Ettle B, Kuhbandner K, Jörg S, Hoffmann A, Winkler J, Linker RA. α-Synuclein deficiency promotes neuroinflammation by increasing Th1 cell-mediated immune responses. J Neuroinflammation. 2016;13(1):201.

72. Guo Y, Chan KH, Lai WH, Siu CW, Kwan SC, Tse HF, et al. Human mesenchymal stem cells upregulate CD1dCD5(+) regulatory B cells in experimental autoimmune encephalomyelitis. Neuroimmunomodulation. 2013;20(5):294-303.
